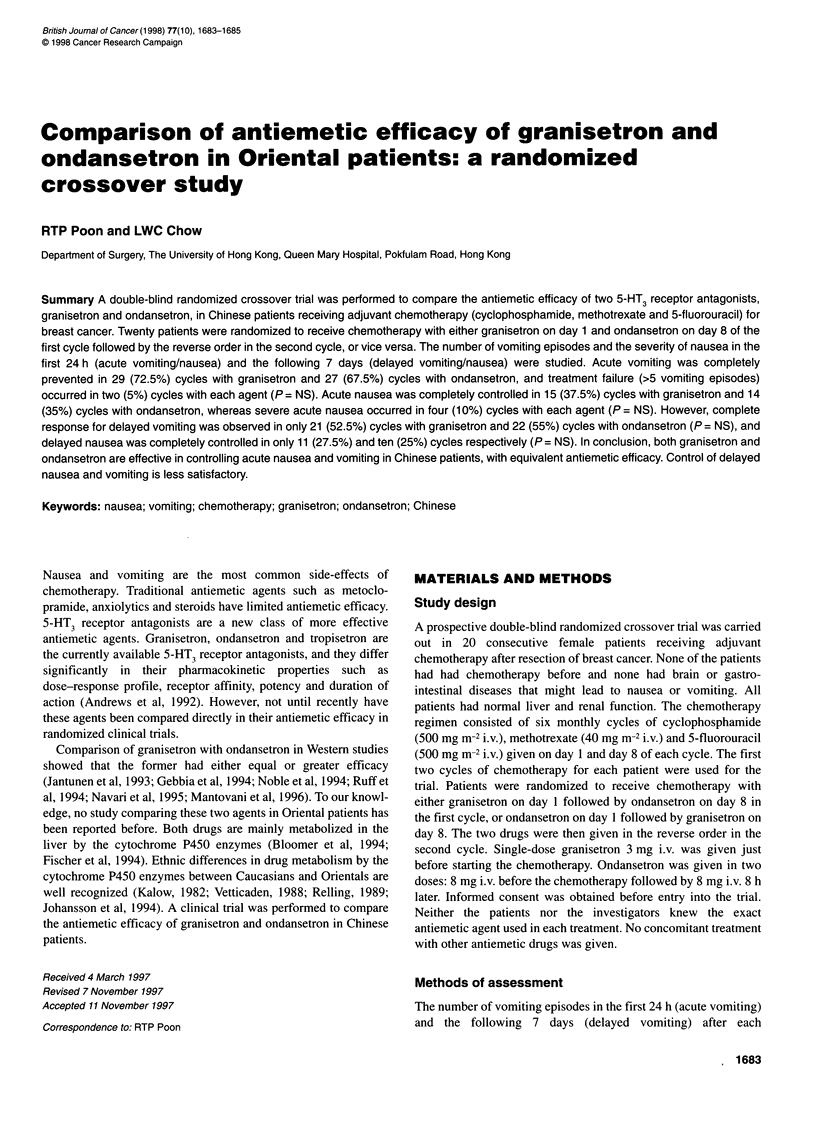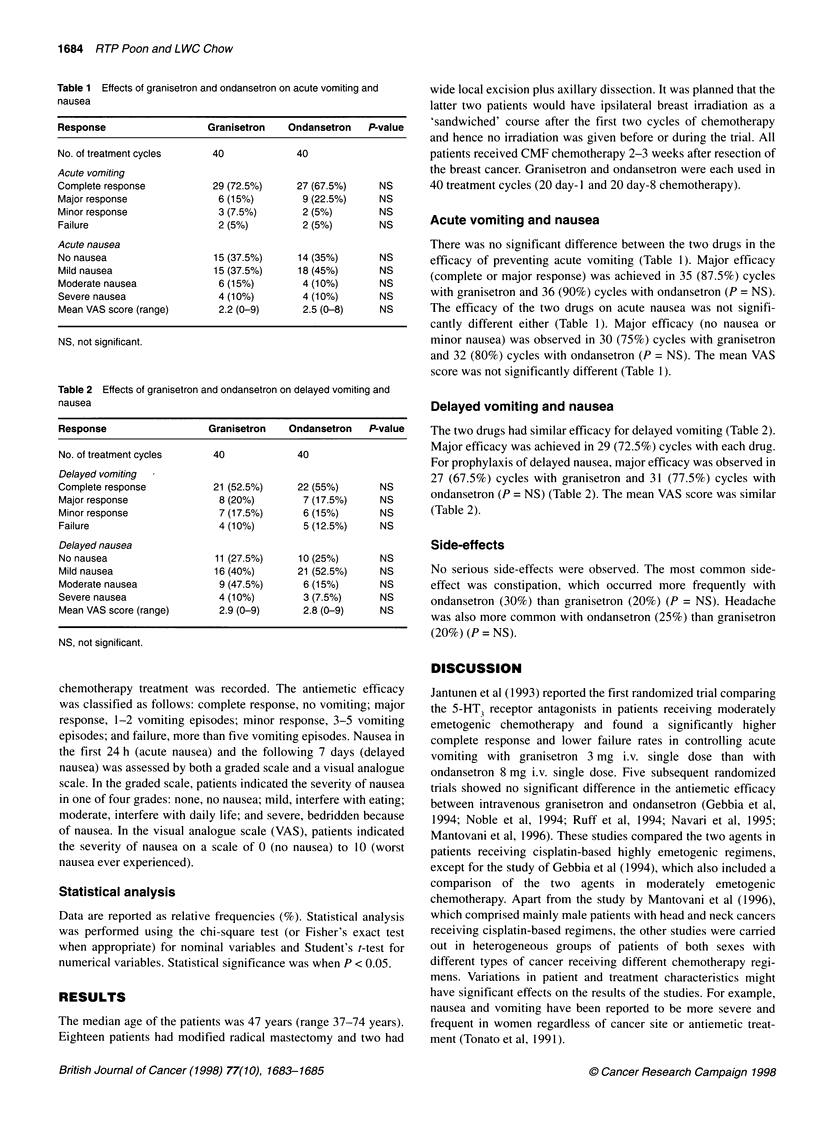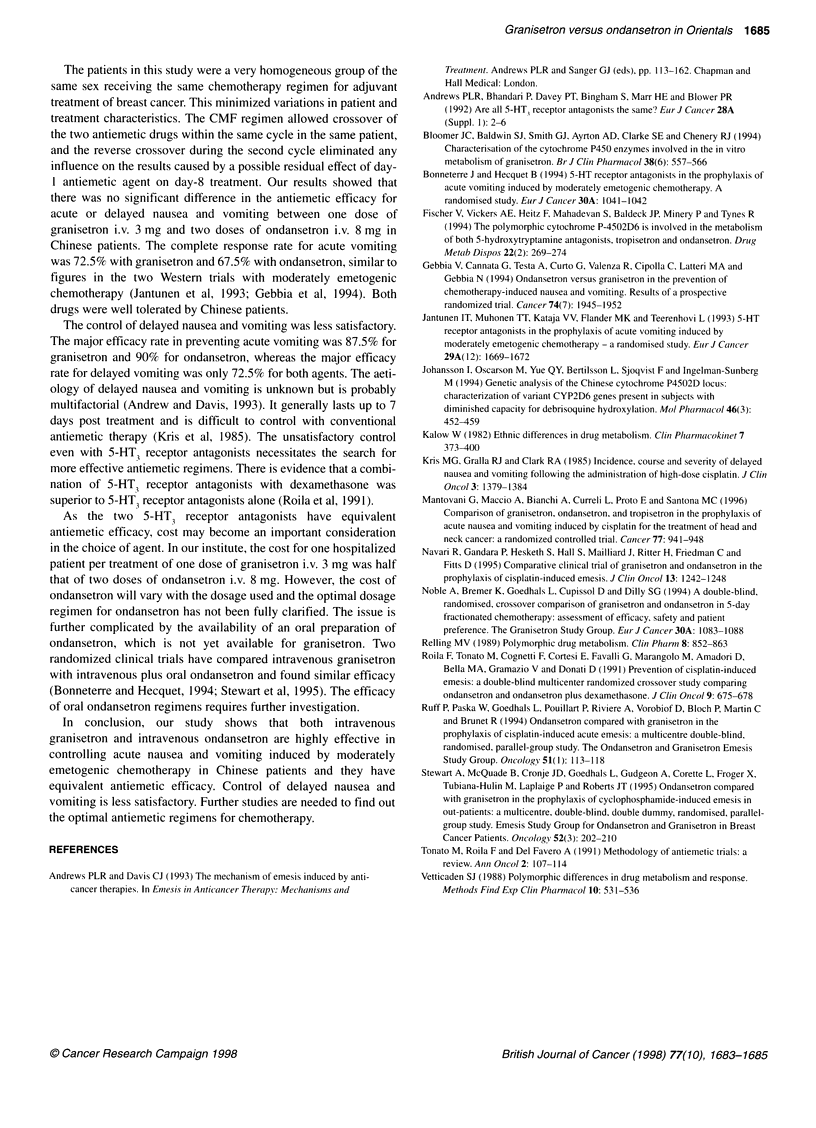# Comparison of antiemetic efficacy of granisetron and ondansetron in Oriental patients: a randomized crossover study.

**DOI:** 10.1038/bjc.1998.277

**Published:** 1998-05

**Authors:** R. T. Poon, L. W. Chow

**Affiliations:** Department of Surgery, The University of Hong Kong, Queen Mary Hospital, Hong Kong.

## Abstract

A double-blind randomized crossover trial was performed to compare the antiemetic efficacy of two 5-HT3 receptor antagonists, granisetron and ondansetron, in Chinese patients receiving adjuvant chemotherapy (cyclophosphamide, methotrexate and 5-fluorouracil) for breast cancer. Twenty patients were randomized to receive chemotherapy with either granisetron on day 1 and ondansetron on day 8 of the first cycle followed by the reverse order in the second cycle, or vice versa. The number of vomiting episodes and the severity of nausea in the first 24 h (acute vomiting/nausea) and the following 7 days (delayed vomiting/nausea) were studied. Acute vomiting was completely prevented in 29 (72.5%) cycles with granisetron and 27 (67.5%) cycles with ondansetron, and treatment failure (>5 vomiting episodes) occurred in two (5%) cycles with each agent (P = NS). Acute nausea was completely controlled in 15 (37.5%) cycles with granisetron and 14 (35%) cycles with ondansetron, whereas severe acute nausea occurred in four (10%) cycles with each agent (P = NS). However, complete response for delayed vomiting was observed in only 21 (52.5%) cycles with granisetron and 22 (55%) cycles with ondansetron (P = NS), and delayed nausea was completely controlled in only 11 (27.5%) and ten (25%) cycles respectively (P = NS). In conclusion, both granisetron and ondansetron are effective in controlling acute nausea and vomiting in Chinese patients, with equivalent antiemetic efficacy. Control of delayed nausea and vomiting is less satisfactory.


*British Journal of Cancer* (1998) **77**(10), 1683–1685
© 1998 Cancer Research Campaign

### Comparison of antiemetic efficacy of granisetron and ondansetron in Oriental patients: a randomized crossover study

**RTP Poon and LWC Chow**

Department of Surgery, The University of Hong Kong, Queen Mary Hospital, Pokfulam Road, Hong Kong

**Summary** A double-blind randomized crossover trial was performed to compare the antiemetic efficacy of two 5-$HT_3$ receptor antagonists, granisetron and ondansetron, in Chinese patients receiving adjuvant chemotherapy (cyclophosphamide, methotrexate and 5-fluorouracil) for breast cancer. Twenty patients were randomized to receive chemotherapy with either granisetron on day 1 and ondansetron on day 8 of the first cycle followed by the reverse order in the second cycle, or vice versa. The number of vomiting episodes and the severity of nausea in the first 24 h (acute vomiting/nausea) and the following 7 days (delayed vomiting/nausea) were studied. Acute vomiting was completely prevented in 29 (72.5%) cycles with granisetron and 27 (67.5%) cycles with ondansetron, and treatment failure (>5 vomiting episodes) occurred in two (5%) cycles with each agent ($P$ = NS). Acute nausea was completely controlled in 15 (37.5%) cycles with granisetron and 14 (35%) cycles with ondansetron, whereas severe acute nausea occurred in four (10%) cycles with each agent ($P$ = NS). However, complete response for delayed vomiting was observed in only 21 (52.5%) cycles with granisetron and 22 (55%) cycles with ondansetron ($P$ = NS), and delayed nausea was completely controlled in only 11 (27.5%) and ten (25%) cycles respectively ($P$ = NS). In conclusion, both granisetron and ondansetron are effective in controlling acute nausea and vomiting in Chinese patients, with equivalent antiemetic efficacy. Control of delayed nausea and vomiting is less satisfactory.

**Keywords:** nausea; vomiting; chemotherapy; granisetron; ondansetron; Chinese

Nausea and vomiting are the most common side-effects of chemotherapy. Traditional antiemetic agents such as metoclopramide, anxiolytics and steroids have limited antiemetic efficacy. 5-$HT_3$ receptor antagonists are a new class of more effective antiemetic agents. Granisetron, ondansetron and tropisetron are the currently available 5-$HT_3$ receptor antagonists, and they differ significantly in their pharmacokinetic properties such as dose–response profile, receptor affinity, potency and duration of action (Andrews et al, 1992). However, not until recently have these agents been compared directly in their antiemetic efficacy in randomized clinical trials.

Comparison of granisetron with ondansetron in Western studies showed that the former had either equal or greater efficacy (Jantunen et al, 1993; Gebbia et al, 1994; Noble et al, 1994; Ruff et al, 1994; Navari et al, 1995; Mantovani et al, 1996). To our knowledge, no study comparing these two agents in Oriental patients has been reported before. Both drugs are mainly metabolized in the liver by the cytochrome P450 enzymes (Bloomer et al, 1994; Fischer et al, 1994). Ethnic differences in drug metabolism by the cytochrome P450 enzymes between Caucasians and Orientals are well recognized (Kalow, 1982; Vetticaden, 1988; Relling, 1989; Johansson et al, 1994). A clinical trial was performed to compare the antiemetic efficacy of granisetron and ondansetron in Chinese patients.

*Received 4 March 1997*
*Revised 7 November 1997*
*Accepted 11 November 1997*

*Correspondence to:* RTP Poon

### MATERIALS AND METHODS

### Study design

A prospective double-blind randomized crossover trial was carried out in 20 consecutive female patients receiving adjuvant chemotherapy after resection of breast cancer. None of the patients had had chemotherapy before and none had brain or gastrointestinal diseases that might lead to nausea or vomiting. All patients had normal liver and renal function. The chemotherapy regimen consisted of six monthly cycles of cyclophosphamide (500 mg $m^{-2}$ i.v.), methotrexate (40 mg $m^{-2}$ i.v.) and 5-fluorouracil (500 mg $m^{-2}$ i.v.) given on day 1 and day 8 of each cycle. The first two cycles of chemotherapy for each patient were used for the trial. Patients were randomized to receive chemotherapy with either granisetron on day 1 followed by ondansetron on day 8 in the first cycle, or ondansetron on day 1 followed by granisetron on day 8. The two drugs were then given in the reverse order in the second cycle. Single-dose granisetron 3 mg i.v. was given just before starting the chemotherapy. Ondansetron was given in two doses: 8 mg i.v. before the chemotherapy followed by 8 mg i.v. 8 h later. Informed consent was obtained before entry into the trial. Neither the patients nor the investigators knew the exact antiemetic agent used in each treatment. No concomitant treatment with other antiemetic drugs was given.

### Methods of assessment

The number of vomiting episodes in the first 24 h (acute vomiting) and the following 7 days (delayed vomiting) after each

**Table 1** Effects of granisetron and ondansetron on acute vomiting and nausea

| Response | Granisetron | Ondansetron | P-value |
|---|---|---|---|
| No. of treatment cycles | 40 | 40 | |
| *Acute vomiting* | | | |
| Complete response | 29 (72.5%) | 27 (67.5%) | NS |
| Major response | 6 (15%) | 9 (22.5%) | NS |
| Minor response | 3 (7.5%) | 2 (5%) | NS |
| Failure | 2 (5%) | 2 (5%) | NS |
| *Acute nausea* | | | |
| No nausea | 15 (37.5%) | 14 (35%) | NS |
| Mild nausea | 15 (37.5%) | 18 (45%) | NS |
| Moderate nausea | 6 (15%) | 4 (10%) | NS |
| Severe nausea | 4 (10%) | 4 (10%) | NS |
| Mean VAS score (range) | 2.2 (0–9) | 2.5 (0–8) | NS |

NS, not significant.

**Table 2** Effects of granisetron and ondansetron on delayed vomiting and nausea

| Response | Granisetron | Ondansetron | P-value |
|---|---|---|---|
| No. of treatment cycles | 40 | 40 | |
| *Delayed vomiting* | | | |
| Complete response | 21 (52.5%) | 22 (55%) | NS |
| Major response | 8 (20%) | 7 (17.5%) | NS |
| Minor response | 7 (17.5%) | 6 (15%) | NS |
| Failure | 4 (10%) | 5 (12.5%) | NS |
| *Delayed nausea* | | | |
| No nausea | 11 (27.5%) | 10 (25%) | NS |
| Mild nausea | 16 (40%) | 21 (52.5%) | NS |
| Moderate nausea | 9 (47.5%) | 6 (15%) | NS |
| Severe nausea | 4 (10%) | 3 (7.5%) | NS |
| Mean VAS score (range) | 2.9 (0–9) | 2.8 (0–9) | NS |

NS, not significant.

chemotherapy treatment was recorded. The antiemetic efficacy was classified as follows: complete response, no vomiting; major response, 1–2 vomiting episodes; minor response, 3–5 vomiting episodes; and failure, more than five vomiting episodes. Nausea in the first 24 h (acute nausea) and the following 7 days (delayed nausea) was assessed by both a graded scale and a visual analogue scale. In the graded scale, patients indicated the severity of nausea in one of four grades: none, no nausea; mild, interfere with eating; moderate, interfere with daily life; and severe, bedridden because of nausea. In the visual analogue scale (VAS), patients indicated the severity of nausea on a scale of 0 (no nausea) to 10 (worst nausea ever experienced).

### Statistical analysis

Data are reported as relative frequencies (%). Statistical analysis was performed using the chi-square test (or Fisher's exact test when appropriate) for nominal variables and Student's *t*-test for numerical variables. Statistical significance was when $P < 0.05$.

### RESULTS

The median age of the patients was 47 years (range 37–74 years). Eighteen patients had modified radical mastectomy and two had wide local excision plus axillary dissection. It was planned that the latter two patients would have ipsilateral breast irradiation as a 'sandwiched' course after the first two cycles of chemotherapy and hence no irradiation was given before or during the trial. All patients received CMF chemotherapy 2–3 weeks after resection of the breast cancer. Granisetron and ondansetron were each used in 40 treatment cycles (20 day-1 and 20 day-8 chemotherapy).

### Acute vomiting and nausea

There was no significant difference between the two drugs in the efficacy of preventing acute vomiting (Table 1). Major efficacy (complete or major response) was achieved in 35 (87.5%) cycles with granisetron and 36 (90%) cycles with ondansetron ($P$ = NS). The efficacy of the two drugs on acute nausea was not significantly different either (Table 1). Major efficacy (no nausea or minor nausea) was observed in 30 (75%) cycles with granisetron and 32 (80%) cycles with ondansetron ($P$ = NS). The mean VAS score was not significantly different (Table 1).

### Delayed vomiting and nausea

The two drugs had similar efficacy for delayed vomiting (Table 2). Major efficacy was achieved in 29 (72.5%) cycles with each drug. For prophylaxis of delayed nausea, major efficacy was observed in 27 (67.5%) cycles with granisetron and 31 (77.5%) cycles with ondansetron ($P$ = NS) (Table 2). The mean VAS score was similar (Table 2).

### Side-effects

No serious side-effects were observed. The most common side-effect was constipation, which occurred more frequently with ondansetron (30%) than granisetron (20%) ($P$ = NS). Headache was also more common with ondansetron (25%) than granisetron (20%) ($P$ = NS).

### DISCUSSION

Jantunen et al (1993) reported the first randomized trial comparing the 5-$HT_3$ receptor antagonists in patients receiving moderately emetogenic chemotherapy and found a significantly higher complete response and lower failure rates in controlling acute vomiting with granisetron 3 mg i.v. single dose than with ondansetron 8 mg i.v. single dose. Five subsequent randomized trials showed no significant difference in the antiemetic efficacy between intravenous granisetron and ondansetron (Gebbia et al, 1994; Noble et al, 1994; Ruff et al, 1994; Navari et al, 1995; Mantovani et al, 1996). These studies compared the two agents in patients receiving cisplatin-based highly emetogenic regimens, except for the study of Gebbia et al (1994), which also included a comparison of the two agents in moderately emetogenic chemotherapy. Apart from the study by Mantovani et al (1996), which comprised mainly male patients with head and neck cancers receiving cisplatin-based regimens, the other studies were carried out in heterogeneous groups of patients of both sexes with different types of cancer receiving different chemotherapy regimens. Variations in patient and treatment characteristics might have significant effects on the results of the studies. For example, nausea and vomiting have been reported to be more severe and frequent in women regardless of cancer site or antiemetic treatment (Tonato et al, 1991).

© Cancer Research Campaign 1998

The patients in this study were a very homogeneous group of the same sex receiving the same chemotherapy regimen for adjuvant treatment of breast cancer. This minimized variations in patient and treatment characteristics. The CMF regimen allowed crossover of the two antiemetic drugs within the same cycle in the same patient, and the reverse crossover during the second cycle eliminated any influence on the results caused by a possible residual effect of day-1 antiemetic agent on day-8 treatment. Our results showed that there was no significant difference in the antiemetic efficacy for acute or delayed nausea and vomiting between one dose of granisetron i.v. 3 mg and two doses of ondansetron i.v. 8 mg in Chinese patients. The complete response rate for acute vomiting was 72.5% with granisetron and 67.5% with ondansetron, similar to figures in the two Western trials with moderately emetogenic chemotherapy (Jantunen et al, 1993; Gebbia et al, 1994). Both drugs were well tolerated by Chinese patients.

The control of delayed nausea and vomiting was less satisfactory. The major efficacy rate in preventing acute vomiting was 87.5% for granisetron and 90% for ondansetron, whereas the major efficacy rate for delayed vomiting was only 72.5% for both agents. The aetiology of delayed nausea and vomiting is unknown but is probably multifactorial (Andrew and Davis, 1993). It generally lasts up to 7 days post treatment and is difficult to control with conventional antiemetic therapy (Kris et al, 1985). The unsatisfactory control even with 5-$HT_3$ receptor antagonists necessitates the search for more effective antiemetic regimens. There is evidence that a combination of 5-$HT_3$ receptor antagonists with dexamethasone was superior to 5-$HT_3$ receptor antagonists alone (Roila et al, 1991).

As the two 5-$HT_3$ receptor antagonists have equivalent antiemetic efficacy, cost may become an important consideration in the choice of agent. In our institute, the cost for one hospitalized patient per treatment of one dose of granisetron i.v. 3 mg was half that of two doses of ondansetron i.v. 8 mg. However, the cost of ondansetron will vary with the dosage used and the optimal dosage regimen for ondansetron has not been fully clarified. The issue is further complicated by the availability of an oral preparation of ondansetron, which is not yet available for granisetron. Two randomized clinical trials have compared intravenous granisetron with intravenous plus oral ondansetron and found similar efficacy (Bonneterre and Hecquet, 1994; Stewart et al, 1995). The efficacy of oral ondansetron regimens requires further investigation.

In conclusion, our study shows that both intravenous granisetron and intravenous ondansetron are highly effective in controlling acute nausea and vomiting induced by moderately emetogenic chemotherapy in Chinese patients and they have equivalent antiemetic efficacy. Control of delayed nausea and vomiting is less satisfactory. Further studies are needed to find out the optimal antiemetic regimens for chemotherapy.

### REFERENCES

Andrews PLR and Davis CJ (1993) The mechanism of emesis induced by anti-cancer therapies. In *Emesis in Anticancer Therapy: Mechanisms and Treatment*. Andrews PLR and Sanger GJ (eds), pp. 113–162. Chapman and Hall Medical: London.

Andrews PLR, Bhandari P, Davey PT, Bingham S, Marr HE and Blower PR (1992) Are all 5-$HT_3$ receptor antagonists the same? *Eur J Cancer* **28A** (Suppl. 1): 2–6

Bloomer JC, Baldwin SJ, Smith GJ, Ayrton AD, Clarke SE and Chenery RJ (1994) Characterisation of the cytochrome P450 enzymes involved in the in vitro metabolism of granisetron. *Br J Clin Pharmacol* **38**(6): 557–566

Bonneterre J and Hecquet B (1994) 5-HT receptor antagonists in the prophylaxis of acute vomiting induced by moderately emetogenic chemotherapy. A randomised study. *Eur J Cancer* **30A**: 1041–1042

Fischer V, Vickers AE, Heitz F, Mahadevan S, Baldeck JP, Minery P and Tynes R (1994) The polymorphic cytochrome P-4502D6 is involved in the metabolism of both 5-hydroxytryptamine antagonists, tropisetron and ondansetron. *Drug Metab Dispos* **22**(2): 269–274

Gebbia V, Cannata G, Testa A, Curto G, Valenza R, Cipolla C, Latteri MA and Gebbia N (1994) Ondansetron versus granisetron in the prevention of chemotherapy-induced nausea and vomiting. Results of a prospective randomized trial. *Cancer* **74**(7): 1945–1952

Jantunen IT, Muhonen TT, Kataja VV, Flander MK and Teerenhovi L (1993) 5-HT receptor antagonists in the prophylaxis of acute vomiting induced by moderately emetogenic chemotherapy – a randomised study. *Eur J Cancer* **29A**(12): 1669–1672

Johansson I, Oscarson M, Yue QY, Bertilsson L, Sjoqvist F and Ingelman-Sunberg M (1994) Genetic analysis of the Chinese cytochrome P4502D locus: characterization of variant CYP2D6 genes present in subjects with diminished capacity for debrisoquine hydroxylation. *Mol Pharmacol* **46**(3): 452–459

Kalow W (1982) Ethnic differences in drug metabolism. *Clin Pharmacokinet* **7** 373–400

Kris MG, Gralla RJ and Clark RA (1985) Incidence, course and severity of delayed nausea and vomiting following the administration of high-dose cisplatin. *J Clin Oncol* **3**: 1379–1384

Mantovani G, Maccio A, Bianchi A, Curreli L, Proto E and Santona MC (1996) Comparison of granisetron, ondansetron, and tropisetron in the prophylaxis of acute nausea and vomiting induced by cisplatin for the treatment of head and neck cancer: a randomized controlled trial. *Cancer* **77**: 941–948

Navari R, Gandara P, Hesketh S, Hall S, Mailliard J, Ritter H, Friedman C and Fitts D (1995) Comparative clinical trial of granisetron and ondansetron in the prophylaxis of cisplatin-induced emesis. *J Clin Oncol* **13**: 1242–1248

Noble A, Bremer K, Goedhals L, Cupissol D and Dilly SG (1994) A double-blind, randomised, crossover comparison of granisetron and ondansetron in 5-day fractionated chemotherapy: assessment of efficacy, safety and patient preference. The Granisetron Study Group. *Eur J Cancer* **30A**: 1083–1088

Relling MV (1989) Polymorphic drug metabolism. *Clin Pharm* **8**: 852–863

Roila F, Tonato M, Cognetti F, Cortesi E, Favalli G, Marangolo M, Amadori D, Bella MA, Gramazio V and Donati D (1991) Prevention of cisplatin-induced emesis: a double-blind multicenter randomized crossover study comparing ondansetron and ondansetron plus dexamethasone. *J Clin Oncol* **9**: 675–678

Ruff P, Paska W, Goedhals L, Pouillart P, Riviere A, Vorobiof D, Bloch P, Martin C and Brunet R (1994) Ondansetron compared with granisetron in the prophylaxis of cisplatin-induced acute emesis: a multicentre double-blind, randomised, parallel-group study. The Ondansetron and Granisetron Emesis Study Group. *Oncology* **51**(1): 113–118

Stewart A, McQuade B, Cronje JD, Goedhals L, Gudgeon A, Corette L, Froger X, Tubiana-Hulin M, Laplaige P and Roberts JT (1995) Ondansetron compared with granisetron in the prophylaxis of cyclophosphamide-induced emesis in out-patients: a multicentre, double-blind, double dummy, randomised, parallel-group study. Emesis Study Group for Ondansetron and Granisetron in Breast Cancer Patients. *Oncology* **52**(3): 202–210

Tonato M, Roila F and Del Favero A (1991) Methodology of antiemetic trials: a review. *Ann Oncol* **2**: 107–114

Vetticaden SJ (1988) Polymorphic differences in drug metabolism and response. *Methods Find Exp Clin Pharmacol* **10**: 531–536

© Cancer Research Campaign 1998